# Neural underpinnings of thematic and taxonomic processing in indirect semantic priming: evidence from the N400 and frontal negativity

**DOI:** 10.1186/s40359-026-04589-0

**Published:** 2026-04-24

**Authors:** Feng Xiao, Na Xiao, Xiuling Liang, Yi Zhong, Jialin Fan, Qingfei Chen

**Affiliations:** 1 School of Psychology, Guizhou Normal University, Guiyang, 550025 China; 2 School of Humanities and Management, Guangdong Medical University, Dongguan, 523808 China; 3 College of Psychology, Shenzhen University, Shenzhen, 518060 China

**Keywords:** Thematic relation, Taxonomic relation, N400, Frontal negativity

## Abstract

**Background:**

Semantic memory includes knowledge of specific categories and their diverse semantic relations, yet the neural mechanisms underlying thematic and taxonomic semantic processing remain controversial.

**Methods:**

We explored the neural correlates of thematic and taxonomic processing via ERP in a lexical decision task. In this task, brand names served as primes, and the extension products served as targets. Five experimental conditions were compared: the core–product condition (e.g., Boeing–airliner), taxonomic condition (Boeing–helicopter), thematic condition (Boeing–suitcase), unrelated condition (Boeing–shirt), and the nonword condition (Boeing–dola) for response balance.

**Results:**

Behavioral results showed significant priming effects (shorter RTs) for core-product, taxonomic, and thematic conditions compared to the unrelated condition. However, N400 analysis revealed that only core-product and thematic conditions showed semantic facilitation (smaller N400), while taxonomic conditions did not differ from unrelated conditions. Critically, frontal negativity (400–550 ms) was smaller for core-product and thematic conditions compared to taxonomic and unrelated conditions.

**Conclusions:**

These findings indicate that thematic relations are more readily retrieved and contextually integrated, whereas taxonomic relations rely on feature-based categorization. The results support the dual-process model of semantic memory and are consistent with the principle of taxonomic primacy with thematic embedding, in which taxonomic structure provides the primary organizational axis while thematic relations are nested within broader contextual networks.

**Supplementary Information:**

The online version contains supplementary material available at 10.1186/s40359-026-04589-0.

## Introduction

Semantic memory is the psychological representation of object categories and the diverse relationships between them, serving as a cornerstone of human cognition [1, 2]. These semantic relationships are critical to many cognitive processes, ranging from language production to similarity judgments and inferential reasoning. According to the dual-process view of semantic organization, this knowledge system is primarily organized along two distinct structural axes: taxonomic relations based on shared features or categories (e.g., car–truck), and thematic relations based on the co-occurrence of entities in specific events or scenarios (e.g., car–gasoline) [2–4]. These axes rely on dissociable neural systems, which aligns with the principle of taxonomic primacy with thematic embedding [2, 5]. Under this framework, taxonomic structure provides the primary organizational axis for semantic memory, while thematic relations are nested within broader contextual networks. Driven by differing feature types and computational demands, these structural axes guide the active semantic processing required during task execution [2]: taxonomic processing relies primarily on feature-based categorization to identify attribute overlap, whereas thematic processing draws on contextual integration and event-based memory retrieval [6, 7]. This theoretical distinction raises a critical empirical question: Do taxonomic and thematic relations rely on distinct neural mechanisms, and at which processing stages do their distinct temporal dynamics emerge?

Despite their functional importance, the neural mechanisms underlying taxonomic and thematic processing remain controversial. Behavioral studies have shown that taxonomic and thematic relations are functionally dissociated, each independently contributing to semantic relatedness [2, 7, 8], in tasks such as relatedness judgments, memory, picture naming, lexical decision, and similarity judgment [9–13], as well as in brand extension [2, 3, 14, 15]. Crucially, fMRI studies has identified the anterior temporal lobe (ATL) and the temporoparietal cortex (TPC) as the respective neural basis for taxonomic and thematic representations [2], suggesting that while the ATL codes stable category structures, the TPC supports the integration of concepts within broader contextual networks [5]. However, ERP studies have failed to reach a consensus on this issue. Early research reported no significant differences between taxonomic and thematic relations [16, 17], whereas later studies reported conflicting patterns. Some researchers observed that thematic relations were processed earlier and elicited greater N1 and P3 components or smaller P2 amplitudes [18, 19]. Others found thematic relations involved more memory processes and elicited a smaller N400/frontal negativity, smaller parietal old–new effect, or increased theta power [20–24]. These contradictory findings highlight the need to clarify whether thematic processing involves earlier automatic activation or more effortful memory retrieval.

Discrepancies in ERP studies on taxonomic and thematic relations stem from differences in concept domains and task properties [2]. First, mixing natural and artificial objects complicates interpretation because these domains can differentially bias taxonomic versus thematic processing [2, 24]. Even studies that restrict materials to artificial objects—using pictures or words—still report inconsistent patterns, indicating that object type alone cannot explain the variability [19, 24]. Beyond this split, living vs. non-living categories and tool vs. non-tool artifacts impose distinct relational constraints (feature overlap vs. action affordances), further shaping component profiles [2, 19, 25]. Second, task properties also modulate ERP effects [2, 26]. For instance, the memory task and the passive listening task are associated respectively with the differences in parietal old–new effect and in theta power [22, 23]. Likewise, categorization/property-verification tends to favor taxonomic similarity, while picture–word or sentence-level integration tasks may enhance thematic facilitation at later stage [27–29]. Hence, the neural mechanisms underlying thematic processing remain poorly understood due to these confounding variables, highlighting the need for further research focused on single concept domains to clarify these effects.

To investigate the neural differences between thematic and taxonomic semantic processing, we used brand extension materials as involve only artificial objects in a lexical decision task. According to the dual-process model of brand extension, consumers evaluate new products through two dissociable cognitive paths [14]. The first path relies on taxonomic relations and is executed through a feature-based comparison process to identify attribute overlap (e.g., Boeing–helicopter). The second path utilizes thematic structures and is characterized by a relation-based integration, involving the retrieval of functional complementarity within shared usage scenarios (e.g., Boeing–suitcase). Behavioral evidence has consistently shown that thematic extensions are processed more rapidly, perceived as less novel, and evaluated more positively, elicit higher purchase intentions than taxonomic ones [6, 14, 15]. However, it remains unclear whether faster thematic processing reflects earlier neural timing [19] or simpler memory retrieval [19, 22]. The event-related potential (ERP) methodology with high temporal resolution is ideally suited to investigate these underlying mechanisms.

Existing ERP studies of brand extension have focused exclusively on taxonomic relations [30–32], leaving thematic processing in this context unexplored. For example, one study used a covert memory task where participants memorized brand names and then viewed product names that were either in-category, similar-category, or out-of-category relative to the brand [31]. The results showed that out-of-category extensions elicited a larger P2 amplitude relative to the in-category and similar-category conditions, while in-category extensions were associated with a smaller N400 amplitude. Accordingly, the authors proposed a two-stage categorization process: the P2 reflected early, low-level similarity processing, and the N400 reflected late, analytic category-based processing. Although the task was not directly related to brand extension evaluation, the use of a covert task likely minimized strategic decision-making, allowing for the detection of automatic semantic categorization. However, it remains unclear whether thematic relations follow a similar two-stage process, highlighting the need to examine both relation types using ERP.

Based on previous studies, we used the high temporal resolution of ERPs to explore the neural dissociation between taxonomic and thematic relations using artificial brand-extension materials. To allow for better comparison with existing literature and our prior studies [14, 20, 24, 31], we used a lexical decision task to investigate semantic relation effects through indirect priming. During the task, participants were asked to decide whether a target was a real word, which was not directly related to brand extension evaluation. In our experiment, brands served as primes (e.g., Boeing) and were paired with five types of targets: Core products (direct priming): The target represented the most prototypical product within the same category as the prime (e.g., Boeing–airliner); Thematically related extensions (indirect priming): The prime and target are linked by event-based or functional connections (e.g., Boeing–suitcase); Taxonomically related extensions (indirect priming): The prime and target share category-based similarity (e.g., Boeing–helicopter); Unrelated extensions: The prime and target are from different categories with no connection (e.g., Boeing–shirt); Nonwords: The target is a nonword for response balance (e.g., dola).

According to the spreading activation model [33], direct semantic priming (e.g., Boeing–airliner) should produce the strongest facilitation, while indirect priming (e.g., Boeing–suitcase or Boeing–helicopter) should yield intermediate effects compared to unrelated pairs. The N400 component is generally considered an index of semantic integration difficulty, with greater amplitude reflecting increased processing effort for semantically incongruent or unexpected stimuli [7, 8, 34]. Based on previous findings [31, 35], we expected that in contrast to the smallest N400 elicited by the core-product condition (direct priming), the unrelated condition (distant extension) would trigger the largest N400, and the thematic/taxonomic conditions (indirect priming) would evoke a median N400.

We focused on the electrophysiological differences between thematic and taxonomic relations, both of which were examined as indirect priming conditions. Behavioral studies have consistently found that thematic extensions are processed faster, judged as less novel, and elicit greater purchase intention than taxonomic extensions [6, 14, 15]. According to the dual-process view and the principle of taxonomic primacy with thematic embedding, taxonomic structure provides the primary organizational axis for semantic memory, while thematic relations are nested within broader contextual networks [2, 5]. Previous ERP research suggests that these behavioral differences may be reflected in both early and late ERP components, corresponding to a two-stage categorization process of brand extension [19, 22, 26, 30, 31, 36]. If thematic relations were processed earlier than taxonomic relations, a significant difference would emerge in the first stage, indexed by early components like N1 and P2, which reflected low-level, similarity-based processing [18–20, 31, 36].

Crucially, if thematic relations were retrieved more easily through controlled semantic processing facilitated by their contextual embedding, differences would appear in the late components, such as the N400 and frontal negativity. Beyond initial access as indexed by N400, the frontal negativity (400–550 ms) is recognized as a critical marker for controlled semantic processing. Previous findings observed that thematic (or productive) relations elicited a significantly reduced frontal negativity compared to taxonomic or unrelated conditions, even when no N400 differences are present [20, 24]. This reduction reflected more efficient scenario-based integration and the recruitment of additional memory resources for constructing cross-category semantic links embedded in event-based networks [20, 24]. Therefore, we hypothesized that while thematic and taxonomic extensions would elicit comparable N400 amplitudes during initial semantic access, thematic extensions would elicit a significantly smaller frontal negativity, reflecting a processing advantage driven by scenario-based integration.

## Methods

### Participants

The required sample size was estimated using G*Power version 3.1 for a repeated-measures ANOVA with four conditions [37]. Parameters were set at *α* = 0.05, power = 0.80, and an assumed medium effect size (*η*^2^ = 0.29), consistent with the frontal negativity effect reported in previous ERP studies [24]. The analysis indicated that a minimum of 18 participants would be sufficient. Nineteen (10 females; *M*_*age*_ = 21.53, *SD* = 1.93) healthy undergraduate students majoring in fields other than psychology or business from Shenzhen University provided written informed consent to participate in the main experiment and received monetary compensation. All participants were right-handed and had normal or corrected-to-normal vision. Participants involved in normative tasks did not take part in the main experiment. The study was approved by the Medical Ethics Committee of Shenzhen University.

### Stimuli

Before the main experiment, 84 well-known brands and their core products were chosen from the “Famous Brands in China” list and previous studies [14], published by the State Trademark Administration of China [31]. Following the methodology of Estes et al. (2012, Study 1B), five marketing graduate students were tasked with generating three types of virtual extensions—taxonomic, thematic, and unrelated—for each brand. Guided by explicit definitions, they were instructed to identify products based either on shared categorical features (taxonomic) or complementary roles in shared usage scenarios (thematic). As a minor refinement, the brand name and its core product were presented simultaneously to provide a clear conceptual anchor for stimulus generation. To ensure experimental control, participants were required to propose only virtual (unreleased) extensions, thereby eliminating potential confounds from prior market knowledge.

Following this, twenty-one participants rated taxonomic and thematic similarity, twenty participants rated brand fit, and twenty-two participants rated familiarity. When rating taxonomic and thematic similarity and brand fit, participants were presented with a core product (e.g., an airliner) and a virtual extension (e.g., a helicopter, a suitcase, or a shirt) simultaneously. They rated the extent to which the items “belong to the same category or have the same features” (taxonomic similarity) and “are related or complement each other” (thematic similarity), as well as the extent to which the virtual extension “fits with the parent brand” (brand fit), using a 7-point scale (1 = not at all, 7 = extremely). For familiarity ratings, participants were presented with all products separately and rated familiarity on the same 7-point scale.

Based on these ratings, we selected 50 brands, their core products, and products that were taxonomically, thematically, or unrelatedly associated with the core products as our final stimuli. Validation analyses revealed significant differences in taxonomic similarity (*F*(2, 98) = 286.65, *p* < .001), thematic similarity *(F*(2, 98) = 255.46, *p* < .001), and brand fit ratings (*F*(2, 98) = 95.95, *p* < .001). As expected, validation analyses confirmed the effectiveness of our stimulus selection. Taxonomic similarity ratings were the highest for taxonomic extensions (5.00 ± 0.10) and the lowest for unrelated extensions (1.93 ± 0.06). Conversely, thematic similarity ratings were the highest for thematic extensions (5.07 ± 0.15) and the lowest for unrelated extensions (1.97 ± 0.09). Taxonomic extensions received intermediate thematic similarity ratings (4.32 ± 0.10), while thematic extensions received intermediate taxonomic similarity ratings (4.14 ± 0.12). *Post-hoc* comparisons using the Bonferroni method confirmed significant differences between all pairwise comparisons (*p*s < 0.001). Importantly, the strength of taxonomic similarity for taxonomic extensions did not differ significantly from the strength of thematic similarity for thematic extensions (*t*(49) = 0.43, *p* = .69), ensuring that both relation types were equally strong in their respective dimensions. Brand fit ratings were equivalent across the taxonomic (4.04 ± 0.12) and thematic conditions (3.98 ± 0.14), with both significantly higher than the unrelated condition (2.12 ± 0.08; *p*s < 0.001). This pattern confirmed that both relation types were perceived as equally plausible brand extensions. Additionally, familiarity ratings showed no significant differences among conditions (taxonomic: 5.17 ± 0.10; thematic: 5.21 ± 0.10; unrelated: 5.19 ± 0.10, *F*(2, 98) < 1), eliminating familiarity as a confounding factor. Similarly, word frequency analysis using the Corpus Word list (http://ccl.pku.edu.cn:8080/ccl_corpus/) revealed no significant differences (taxonomic: 61.5 ± 24.00; thematic: 90.06 ± 20.88; unrelated: 75.24 ± 18.76, *F*(2, 98) < 1), ensuring lexical properties were controlled across conditions.

All brand names were selected from a pool of well-known international and Chinese brands, and were pre-rated by Chinese participants for familiarity, perceived fit, and relatedness. To aid interpretability for international readers, Table S1 provides the full stimulus list with English glosses and notes on potential culture-specific associations (e.g., cases where primes like Boeing may cue different extensions across cultures). We restrict our theoretical claims to the artifact domain sampled here and view cross-cultural generalization as an empirical question for future research.

### Experimental design

We used a lexical decision task in which brands served as primes and products served as targets. The independent variable (within-subjects) was semantic relation with four levels: (1) core-product (direct priming, e.g., Boeing–airliner), (2) taxonomic extension (indirect priming, e.g., Boeing–helicopter), (3) thematic extension (indirect priming, e.g., Boeing–suitcase), and (4) unrelated extension (e.g., Boeing–shirt). Additionally, a nonword condition (e.g., Boeing–dola) was incorporated into the lexical decision task to balance ‘word’ and ‘nonword’ responses but was excluded from the subsequent semantic ERP statistical analysis. Both behavioral measures (accuracy and reaction time) and ERP data were recorded for each target response.

The experiment comprised a total of 600 trials: 50 core product trials, 50 taxonomic trials, 50 thematic trials, 150 unrelated trials, and 300 nonword trials. Trial order was randomized for each participant, and no fixed block structure was imposed. The unequal trial distribution served methodological purposes: the high nonword proportion (50%) maintained task engagement and prevented automatic “word” responses, while the larger number of unrelated trials (150 vs. 50 per related condition) established a robust baseline for assessing semantic priming effects. Despite unequal trial counts, each condition provided sufficient trials (≥ 50) for stable ERP averaging after artifact rejection.

### Procedure

To ensure that all participants were familiar with these brands and to eliminate the impact of differential familiarity, we presented all brands and their corresponding core products to participants at the beginning of the experiment. We then provided task instructions and asked participants to repeat the instructions in their own words to confirm comprehension. Subsequently, we familiarized participants with the procedure through twenty practice trials.

**Fig. 1:**
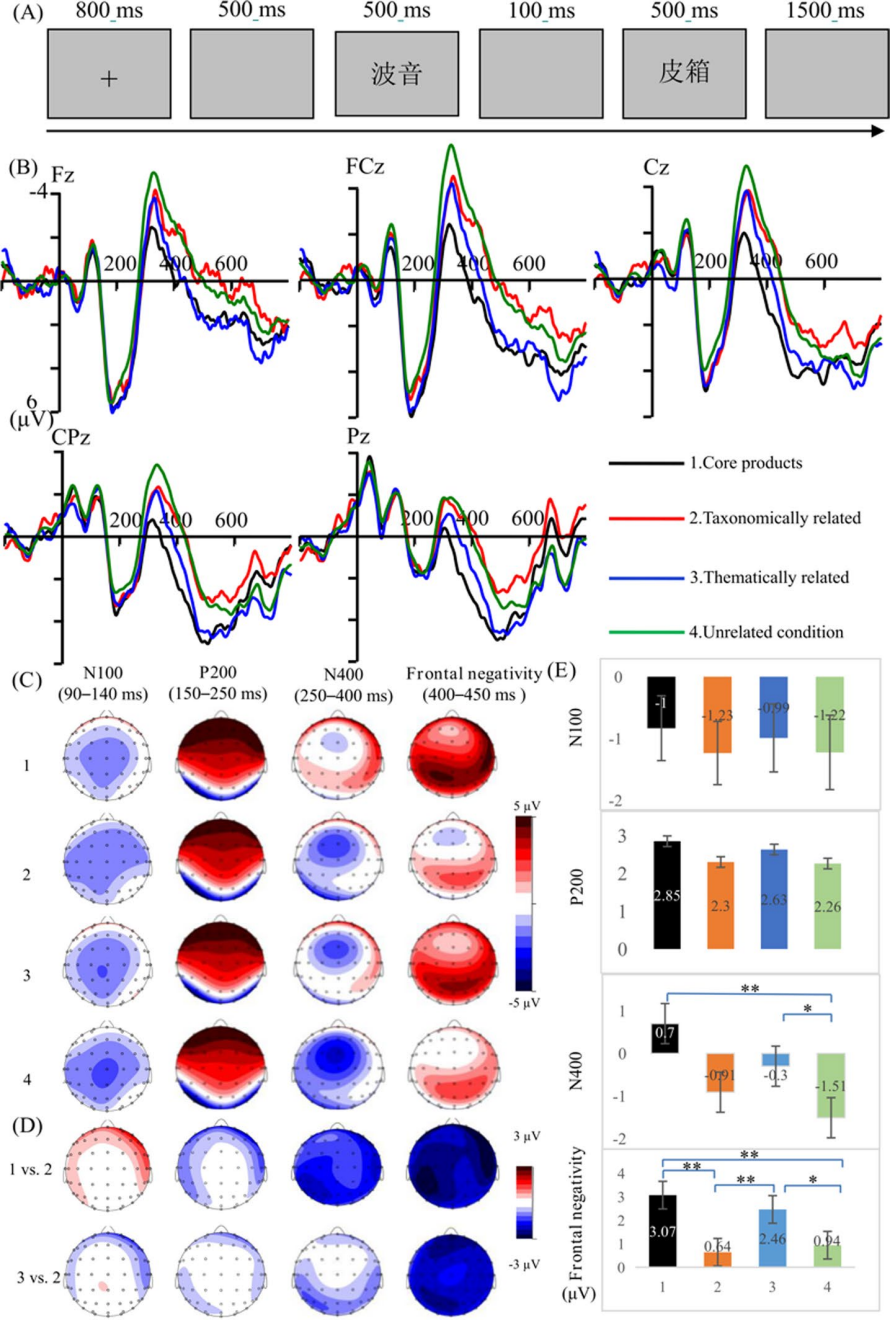
**A** Experimental procedures for the lexical decision task; **B** The original potentials in different conditions in Fz, FCz, Cz, CPz and Pz; **C** Topography of original potentials in different conditions; **D** Differences topographies for core products vs. taxonomically related condition, and for taxonomically related vs. thematically related condition; **E** Grand average ERP amplitude in the N100, P200, N400, and frontal negativity on all 15 analyzed electrodes

As shown in Fig. 1A, each trial began with an 800 ms black fixation cross “+” presented at the center of a gray background. This was followed by a gray blank screen that appeared for 500 ms, then a brand name appeared at the center for 500 ms. Next, a gray screen was displayed for 100 ms, after which the target appeared centrally for 500 ms. Each trial concluded with a gray screen presented for 1500 ms. Participants were instructed to judge whether the target was a real word as quickly and accurately as possible by pressing either the “F” or “J” key with their left or right index finger, respectively. Response key assignments (word/nonword) were counterbalanced across participants.

### ERP recordings and data analysis

EEG data were recorded using a 64-channel Brain Products system (Brain Products GmbH, Munich, Germany; passband: 0.05–100 Hz, sampling rate: 500 Hz) based on the extended 10/20 system. The ground electrode was located at the medial frontal site (Fz), and the left and right mastoids were used as reference electrodes [38, 39]. Electrooculogram (EOG) was recorded from an electrode placed infraorbitally below the left eye, and all channel impedances were maintained below 10 kΩ. EEG data were preprocessed off-line using Brain Vision Analyzer 2.0. Artifacts (e.g., blinks, eye movements) were removed using independent component analysis (ICA) for ocular correction [40, 41]. Independent components (ICs) associated with eye movements were identified by their large EOG channel contributions and frontal scalp distributions. All data were visually inspected to identify bad epochs and were rejected if necessary. The signal was filtered using a 0.1 to 35 Hz digital band-pass filter for offline analysis. Trials with EOG artifacts (mean EOG voltages exceeding ± 80 µV) or artifacts from amplifier clipping, muscle potentials, or peak-to-peak deflections exceeding ± 80 µV were excluded from analysis.

Event-related potentials (ERPs) were computed by averaging time-locked waveforms to the onsets of the targets for each participant and condition. EEG epochs were segmented into 1.0-s time windows, including a 0.2-s pre-stimulus interval used as the baseline for amplitude calculations. Following previous studies [20, 24, 26, 31], repeated-measures ANOVAs were conducted on mean amplitudes within 150–250 ms, 250–400 ms, and 400–550 ms time windows, with experimental condition, laterality (3 levels: left, midline, right) and frontality (5 levels: frontal [left–F3, midline–Fz, right–F4]; fronto–central [left–FC3, midline–FCz, right–FC4]; central [left–C3, midline–Fz Cz, right–C4]; centro-parietal [left–CP3, midline–CPz, right–CP4]; and parietal [left–P3, midline–Pz, right–P4]) as within-subjects factors. For all analyses, degrees of freedom were corrected for sphericity violations using the Greenhouse-Geisser correction, and Bonferroni corrections were applied for multiple comparisons.

## Results

### Behavioral results

We conducted repeated-measures ANOVAs on accuracy and reaction times (RTs). Analysis revealed a main effect of condition on RTs, *F*(3, 54) = 39.51, *p* < .001, *η*_p_² = 0.69, *power* = 1.00. Pairwise comparisons showed significant differences between all condition pairs (*p*s < 0.05), except for the comparison between the taxonomic and the thematic conditions (*p* > .90). To assess semantic priming effects, we compared each related condition with the unrelated condition using planned paired contrasts. Results confirmed significant priming effects: RTs were significantly shorter for the core–product condition (534 ± 22 ms, *p* < .001), the taxonomic condition (573 ± 24 ms, *p* < .05), and the thematic condition (570 ± 23 ms, *p* < .001), compared to the unrelated condition (594 ± 23 ms).

For accuracy, we found a significant main effect of condition, *F*(3, 54) = 20.10, *p* < .001, *η*_p_² = 0.53, power = 1.00. *Post-hoc* pairwise comparisons revealed that accuracy for the core-product condition (99 ± 1%) was significantly higher than that for the taxonomic condition (94 ± 1%, *p* < .001), the thematic condition (96 ± 1%, *p* = .01), and the unrelated condition (92 ± 1%, *p* < .001). Additionally, accuracy for the thematic condition was significantly higher than that for the unrelated condition (*p* = .010). No other significant differences were observed.

### ERP Results

#### N100 (90–140 ms) and P200 (150–250 ms)

As shown in Table 1; Fig. 1, there was no significant effect of condition on N100 or P200 amplitude. Additionally, significant main effects of frontality and laterality were observed, indicating larger N100 peaking over the parietal-central sites (ps < 0.031), and greater P200 amplitudes at frontal, fronto-central, and midline sites (ps < 0.035). Unlike previous studies reporting N100 and P200 modulation in semantic processing (e.g., brand extension categorization [31]), no N100 or P200 differences were found here, possibly due to the lexical decision task’s characteristics or the use of word stimuli rather than pictures.

**Table 1:** Three-way repeated-measures ANOVA of mean amplitudes to assess the processing of thematic and taxonomic relations. The significant effects are written in bold. C: Condition; F: Frontality; L: Laterality

		Main effect			Interaction			
Window	value	F	L	C	F×L	F×C	L×C	F×L×C
N100 (90–140 ms)	*F*	0.98	**5.11**	0.47	**5.35**	0.99	0.84	0.92
	*P*	0.353	**0.012**	0.688	**0.004**	0.413	0.474	0.462
	*η*_*p*_^*2*^	0.05	**0.22**	0.03	**0.23**	0.05	0.05	0.05
	*Power*	0.17	**0.87**	0.13	**0.89**	0.27	0.22	0.29
P200 (150–250 ms)	*F*	**57.06**	**7.18**	1.06	**13.08**	0.73	1.44	0.75
	*P*	**< 0.001**	**0.003**	0.371	**< 0.001**	0.586	0.236	0.569
	*η*_*p*_^*2*^	**0.76**	**0.29**	0.06	**0.42**	0.04	0.07	0.04
	*Power*	**0.99**	**0.91**	0.27	**0.99**	0.23	0.40	0.24
N400 (250–400 ms)	*F*	1.57	**7.25**	**6.98**	2.58	1.17	1.04	1.05
	*P*	0.228	**0.003**	**0.001**	0.053	0.330	0.381	0.390
	*η*_*p*_^*2*^	0.08	**0.29**	**0.28**	0.13	0.06	0.05	0.06
	*Power*	0.25	**0.91**	**0.94**	0.65	0.35	0.25	0.33
Frontal Negativity (400–550 ms)	*F*	**5.68**	0.96	**9.56**	**3.01**	0.75	0.39	1.00
	*P*	**0.019**	0.371	**< 0.001**	**0.033**	0.558	0.770	0.421
	*η*_*p*_^*2*^	**0.24**	0.05	**0.35**	**0.14**	0.04	0.02	0.05
	*Power*	**0.70**	0.18	**0.99**	**0.71**	0.22	0.12	0.33

#### N400 (250–400 ms)

The analyses showed significant effect of condition on N400 amplitude. *Post-hoc* pairwise comparisons showed larger N400 amplitude for the unrelated condition (-1.51 µV) relative to the core-product condition (0.70 µV, *ps* < 0.05) and the thematic condition (-0.30 µV, *ps* < 0.05), but not relative to the taxonomic condition (-0.91 µV, *p* > .90) (see Fig. 1D). Furthermore, more negative N400 were found for the taxonomic condition relative to the core-product condition (*ps* < 0.05). However, no significant difference was observed between the taxonomic and the thematic conditions (*p* > .90). Additionally, a significant main effect of laterality was found, indicating larger N400 amplitudes at left and midline region than right hemisphere (*p*s < 0.056).

#### Frontal Negativity (400–550 ms)

Analysis showed a significant effect of condition on frontal negativity. *Post-hoc* pairwise comparisons revealed that frontal negativity was less negative for the core-product condition (3.07 µV, *p*s < 0.01) and the thematic condition (2.46 µV, *p*s < 0.05) compared to the taxonomic (0.64 µV) and unrelated conditions (0.94 µV). Additionally, a significant interaction effect of frontality×laterality was observed, showing that the frontal negativity was larger at left frontal and fronto-central sites than parietal regions (*p*s < 0.059).

## Discussion

In this ERP study, we aimed to examine the neural dissociation between taxonomic and thematic relations using a lexical decision task. We controlled brand similarity, perceived fit, and familiarity across all conditions, ensuring that semantic relation types, not confounding variables, drove the observed effects. This approach minimized interference from pre-existing brand associations. Behavioral data showed that participants responded fastest to core products, slower to taxonomically and thematically related extensions, and slowest to unrelated extensions. These results were consistent with research on direct and indirect semantic priming, which found larger priming effects for direct relative to indirect priming [35, 42, 43]. In the present study, semantic priming for core products was direct (e.g., Boeing-airliner), whereas semantic priming for the thematic and taxonomic conditions was indirect (e.g., Boeing-helicopter/suitcase), resulting in a larger priming effect for core products compared to thematic and taxonomic conditions. However, no significant behavioral difference was found between the thematic and taxonomic conditions. Overall, these findings were consistent with the spreading activation model, demonstrating that primed content increases the accessibility of associated content [33, 44].

In our ERP results, we found no significant differences across conditions for the early components, specifically the N100 and P200. This result diverged from previous ERP studies that had reported early modulation in semantic or brand-extension tasks, where out-of-category items often elicited greater P200 amplitudes [31, 45]. A primary explanation for this discrepancy likely lies in the nature of the task: As the inductive reasoning task required participants to actively identify and judge logical links between premises and conclusions, taxonomic relations elicited larger P2 amplitudes than thematic relations [18]. Similarly, while thematic relations elicited greater N1 and P3 components, this study utilized pictorial stimuli in picture-naming paradigms [19]. In contrast, our study not only emphasized implicit semantic priming rather than explicit categorization, but also employed purely word stimuli, which minimized early perceptual differences and attention capture. Thus, the absence of early component effects in the current study suggests that early perceptual processing plays a less prominent role than memory retrieval and semantic integration in brand-extension-related semantic priming.

We found that target type modulated N400 amplitude. Specifically, the N400 was less negative for core products (direct priming), intermediate for taxonomically and thematically related extensions (indirect priming), and most negative for unrelated extensions. These findings aligned with previous studies on semantic relations [34, 35], which had shown that N400 amplitude increased with greater semantic incongruity, supporting that larger N400 amplitudes were typically associated with increased semantic processing effort [7, 8, 30]. Importantly, brand similarity, perceived fit, and familiarity were well controlled across conditions, ruling out alternative explanations. Notably, no N400 difference was observed between thematic and taxonomic extensions. This suggests that both relation types require similar levels of semantic integration effort at the initial semantic access stage, which fails to support the view of an early processing advantage for thematic relations [20, 24, 45–47]. The view was also supported by the absence of any early component effects, typically linked to early perceptual processing, even when lexical and associative factors are controlled. Hence, the semantic integration stage indexed by the N400 suggests that thematic and taxonomic relations impose a similar processing load.

Although similar semantic priming effects (RTs and N400 effects) were observed for both indirect priming conditions, thematically related extensions and core products elicited smaller frontal negativities than taxonomically related and unrelated extensions in the 400–550 ms time window. This frontal negativity shared spatial and temporal characteristics with the FN400 in recognition memory studies [7, 48–50] and the Late Negative Component (LNC) in reasoning tasks [20, 24, 47], reflecting controlled semantic processing mechanisms within the dual-process framework. According to spreading-activation theory, taxonomic extensions should show stronger priming than thematic extensions due to shared categorical features, while thematic extensions stored in distant semantic nodes should produce larger frontal negativities [33, 44]. However, our findings contradicted this expectation, showing decreased frontal negativity for thematic extensions, consistent with previous direct-to-indirect semantic priming studies [20, 24, 45] but inconsistent with spreading-activation predictions. This pattern suggests that thematic processing involves a fundamentally different integration mechanism that transcends simple nodal distance, supporting the dual-process model where contextual integration and feature-based comparison serve as dissociable systems [20–24]. These results indicate that thematic–taxonomic distinctions operate primarily at late integration stages rather than early perceptual or lexical stages.

Thematic relations in this study were in line with the productive relation (e.g., bee–honey) in our previous ERP work [20] and elicited a smaller frontal negativity than taxonomic relations (e.g., dog–cat). This decreased frontal negativity effect found in thematic relations indicates that, while taxonomic processing relies on an analytic comparison of internal feature overlap, thematic processing depends on the efficiency of scenario-based or functional integration [2, 4]. The same conclusion can be supported by the representational account of taxonomic primacy with thematic embedding [5], according to which taxonomic structure constitutes the primary organizational axis while thematic information is embedded within broader scene/event networks. Thus, although thematic relations rely on longer semantic pathways, they are more readily retrieved and efficiently integrated when contextualized within a shared scenario [5]. Together, the frontal negativity pattern and prior evidence indicate that thematic relations rely more on controlled scenario-based integration as a distinct mechanism from feature-based categorization, and frontal negativity serves as a late-stage marker of this dual-process coordination when behavior and N400 are comparable.

Similarly, the frontal negativity observed in this study parallels the LNC in reasoning studies, sharing similar timing, polarity, and a characteristic anterior (frontal/fronto-central) scalp distribution. The LNC is typically elicited during complex conditional or categorical reasoning and is thought to index two key processes: inhibition of cognitive conflict and integration of semantic relations [20, 24, 51, 52]. It has also been associated with relational integration, namely the ability to construct higher-order semantic links across multiple cues [53, 54]. Within the dual-process framework, the reduced frontal negativity for thematic relations we observed here suggests stronger engagement of these integrative operations. Unlike taxonomic processing, which relies on direct feature overlap, thematic processing involves integrating the prime with related semantic representations and inferring contextual connections to the target, thereby constructing cross-category semantic links [5, 20, 52]. This distinct mechanism allows thematic relations to be more readily retrieved and efficiently integrated when contextualized within shared usage scenarios. Therefore, the observed frontal-negativity modulation aligns with the view that thematic activation utilizes controlled contextual integration rather than mere feature-based categorization [20, 24, 50], serving as a late-stage marker of the functional dissociation between the two semantic systems.

We observed a dissociation between behavioral and ERP measures. Although behavioral measures provide a holistic view of semantic facilitation, ERP components allow dissection of temporal stages, revealing nuances not captured in RTs. Behaviorally, direct priming yielded greater facilitation than indirect priming, and RTs did not differ reliably between the thematic and taxonomic conditions. This pattern was broadly consistent with spreading-activation accounts [33, 44] and was anticipated given an indirect-priming lexical decision task with materials matched on relatedness, fit, and familiarity [7]. In speeded tasks, responses primarily tracked global semantic overlap rather than relational structure, rendering small relational advantages unlikely to emerge in RTs. By contrast, within pre-specified windows, the ERPs showed comparable N400 demands across the two relations but a robust dissociation in the late frontal negativity (400–550 ms), with an anterior distribution consistent with later context integration/reevaluation [24, 50]. We interpreted this frontal negativity effect functionally (on timing/topography) rather than as evidence for distinct generators. Thus, in this paradigm, neural sensitivity exceeds behavioral sensitivity, placing the primary thematic–taxonomic difference at a late integration stage rather than initial access.

Our study replicated the indirect semantic priming effect behaviorally and revealed neural mechanisms underlying thematic relations distinct from taxonomic relations. The results provide robust support for the dual-process model of semantic memory [14, 15], where taxonomic processing relies on the analytical comparison of internal features, while thematic processing draws on dynamic scenario-based integration, allowing thematic links to be more readily retrieved and efficiently integrated. Building on the similar N400 patterns across relation types, the reduced frontal negativity highlights a functional dissociation in cognitive resource allocation and integration strategies. This finding is consistent with the principle of taxonomic primacy and thematic embedding [5], which posits that while taxonomic structure serves as the primary organizational axis, thematic relations are nested within broader contextual networks that facilitate efficient relational integration. While our lexical decision task was not directly related to brand extension evaluation, it successfully isolated semantic processing effects, minimizing confounds from prior brand knowledge or emotional evaluation [7, 42]. These findings suggest that thematic relations facilitate brand concept activation through scenario-based associations, offering a neural explanation for why thematic links often enhance brand extension acceptance [6, 14]. Future research could further test these mechanisms using multimodal stimuli or alternative semantic tasks (such as memory tasks) to clarify early-stage processing differences, including the role of early ERP components (P2, N1, P3).

## Conclusions

This study used ERPs to examine the neural mechanisms of taxonomic and thematic relations to clarify the “automatic versus effortful retrieval” debate. Behaviorally, core products elicited the fastest responses and highest accuracy, while both taxonomic and thematic extensions showed indirect priming without significant N400 differences. Crucially, core products and thematic extensions evoked reduced frontal negativity compared to taxonomic and unrelated conditions, indicating that thematic processing draws on dynamic scenario-based integration, whereas taxonomic processing relies on the analytical comparison of internal features. Overall, the findings support the dual-process model of semantic memory and further align with the principle of taxonomic primacy with thematic embedding, in which taxonomic relations provide the primary organizational axis of semantic memory, and thematic relations are embedded within broader contextual and event-based networks. These results provide new evidence for the distinct but complementary cognitive roles of taxonomic and thematic relations, with implications for understanding semantic organization and applications such as brand extension.

## Supplementary Information

Supplementary Material 1.

## Data Availability

The data will be available from the corresponding author upon reasonable request.

## Abbreviations

ERP: Event-related potential
ACC: Accuracy
RT: Response times
N400: Negativity 400(an ERP component occurring around 400 ms, associated with semantic processing)
FN400: Frontal N400
P200: Positive 200 (an ERP component occurring around 200 ms, associated with perceptual and attentional processes)
LNC: Late Negative Component
