# Supplementary material for: Neural underpinnings of thematic and taxonomic processing in indirect semantic priming: evidence from the N400 and frontal negativity
Source: BMC Psychol. 2026 Apr 24;14:773. doi: 10.1186/s40359-026-04589-0 (PMC13196190; doi:10.1186/s40359-026-04589-0)
Supplement: Supplementary file 1 — Supplementary Material 1. [file 40359_2026_4589_MOESM1_ESM.docx]

| S1. experimental materials | | | | | | | | |
| --- | --- | --- | --- | --- | --- | --- | --- | --- |
| No. | Brand  (CN) | Brand  (EN) | Core  Product  (CN) | Core  Product  (EN) | Taxonomically related  (CN) | Thematically related(EN) | Unrelated  condition  (CN) | Unrelated condition  (EN) |
| 1 | 波音 | Boeing | 客机 | passenger aircraft | 飞船 | spacecraft | 衬衣 | dress shirt |
| 2 | 联想 | Lenovo | 电脑 | computer | 手表 | smartwatch | 手套 | gloves |
| 3 | 万宝路 | Marlboro | 香烟 | cigarettes | 旱烟 | loose-leaf tobacco | 钥匙 | keys |
| 4 | 豪爵 | Haojue | 摩托 | motorcycle | 三轮 | three-wheeler | 铅笔 | pencil |
| 5 | 星巴克 | Starbucks | 咖啡 | coffee | 红酒 | red wine | 桅杆 | mast |
| 6 | 丰田 | Toyota | 汽车 | automobile | 摩托 | motorcycle | 字帖 | copybook |
| 7 | 优酷 | Youku | 视频 | online video | 图书 | books | 钢笔 | fountain pen |
| 8 | 雪花 | Snow | 啤酒 | beer | 可乐 | cola | 轮胎 | tire |
| 9 | 德芙 | Dove | 巧克力 | chocolate | 果冻 | jelly | 锄头 | hoe |
| 10 | 沃尔玛 | Walmart | 超市 | supermarket | 街市 | street market | 浴室 | bathroom |
| 11 | 肯德基 | KFC | 炸鸡 | fried chicken | 寿司 | sushi | 婚纱 | wedding dress |
| 12 | 迪斯尼 | Disney | 游乐园 | amusement park | 博物馆 | museum | 中药 | traditional Chinese medicine |
| 13 | 希尔顿 | Hilton | 酒店 | hotel | 别墅 | villa | 气泵 | air pump |
| 14 | 乐百氏 | Robust | 矿泉水 | mineral water | 白酒 | liquor | 气泵 | air pump |
| 15 | 汇源 | Huiyuan | 果汁 | juice | 奶茶 | milk tea | 烫斗 | iron |
| 16 | 真彩 | TrueColor | 文具 | stationery | 玩具 | toys | 冰鞋 | ice skates |
| 17 | 鲁花 | Luhua | 粮油 | cooking oil | 鸡精 | chicken bouillon | 书本 | book |
| 18 | 意尔康 | Yearcon | 皮鞋 | leather shoes | 布鞋 | canvas shoes | 泡菜 | kimchi |
| 19 | 海天 | Haitian | 酱油 | soy sauce | 酱菜 | pickles | 雨伞 | umbrella |
| 20 | 碧螺春 | Biluochun | 茶叶 | tea | 蜂蜜 | honey | 电瓶 | battery |
| 21 | 永和 | Yonghe | 豆浆 | soy milk | 啤酒 | beer | 床垫 | mattress |
| 22 | 雅兰 | Airland | 床垫 | mattress | 地毯 | carpet | 牛排 | steak |
| 23 | 统一 | Uni-President | 泡面 | instant noodles | 汉堡 | hamburger | 图册 | picture album |
| 24 | 力士 | Lux | 香皂 | soap | 洗衣粉 | detergent | 瓜子 | sunflower seeds |
| 25 | 小肥羊 | Little Sheep | 火锅 | hot pot | 快餐 | fast food | 琴行 | music store |
| 26 | 黑鹰 | Black Hawk | 直升机 | helicopter | 客机 | passenger aircraft | 电视 | television |
| 27 | 益达 | Extra | 口香糖 | chewing gum | 方糖 | sugar cube | 乐器 | musical instrument |
| 28 | 必胜客 | Pizza Hut | 披萨 | pizza | 汉堡 | hamburger | 教材 | textbook |
| 29 | 斯伯丁 | Spalding | 篮球 | basketball | 台球 | billiards | 陈醋 | mature vinegar |
| 30 | 牛栏 | Niulan | 奶粉 | milk powder | 果粉 | fruit powder | 画笔 | paint brush |
| 31 | 保时捷 | Porsche | 跑车 | sports car | 摩托 | motorcycle | 菜汤 | soup |
| 32 | 维维 | VV | 豆奶 | soy milk | 咖啡 | coffee | 皮箱 | suitcase |
| 33 | 美利达 | Merida | 单车 | bicycle | 汽车 | car | 熨斗 | iron |
| 34 | 百事 | Pepsi | 可乐 | cola | 啤酒 | beer | 熨斗 | iron |
| 35 | 大白兔 | White Rabbit | 奶糖 | milk candy | 果冻 | jelly | 鞋油 | shoe polish |
| 36 | 麦当劳 | McDonald's | 汉堡 | hamburger | 寿司 | sushi | 剧本 | script |
| 37 | 茅台 | Moutai | 白酒 | liquor | 红酒 | red wine | 皮鞋 | leather shoes |
| 38 | 晨光 | M&G | 文具 | stationery | 乐器 | musical instrument | 酱油 | soy sauce |
| 39 | 派克 | Parker | 钢笔 | fountain pen | 画笔 | paint brush | 西装 | suit |
| 40 | 太太乐 | Totole | 鸡精 | chicken bouillon | 陈醋 | mature vinegar | 皮靴 | leather boots |
| 41 | 波司登 | Bosideng | 羽绒服 | down jacket | 西服 | suit | 蜂蜜 | honey |
| 42 | 老干妈 | Lao Gan Ma | 辣椒 | chili sauce | 鸡精 | chicken bouillon | 泡面 | instant noodles |
| 43 | 王老吉 | Wanglaoji | 凉茶 | herbal tea | 汽水 | soda | 眼镜 | glasses |
| 44 | 蒙牛 | Mengniu | 牛奶 | milk | 豆浆 | soy milk | 地毯 | carpet |
| 45 | 洽洽 | Qiaqia | 瓜子 | sunflower seeds | 红酒 | red wine | 鞋油 | shoe polish |
| 46 | 海飞丝 | Head & Shoulders | 洗发水 | shampoo | 皂液 | soap liquid | 水饺 | dumplings |
| 47 | 红牛 | Red Bull | 饮料 | energy drink | 药酒 | medicinal liquor | 轮胎 | tire |
| 48 | 好利来 | Holiland | 蛋糕 | cake | 汉堡 | hamburger | 主板 | motherboard |
| 49 | 李维斯 | Levi's | 牛仔裤 | jeans | 西装 | suit | 帐篷 | tent |
| 50 | 同仁堂 | Tong Ren Tang | 中药 | traditional Chinese medicine | 西药 | Western medicine | 医院 | hospital |

Note: CN = Chinese name; EN = English name.
